# Manipulation of charge transfer and transport in plasmonic-ferroelectric hybrids for photoelectrochemical applications

**DOI:** 10.1038/ncomms10348

**Published:** 2016-01-12

**Authors:** Zhijie Wang, Dawei Cao, Liaoyong Wen, Rui Xu, Manuel Obergfell, Yan Mi, Zhibing Zhan, Nasori Nasori, Jure Demsar, Yong Lei

**Affiliations:** 1 Institut für Physik & IMN MacroNano (ZIK), Technische Universität Ilmenau, 98693 Ilmenau, Germany; 2 Key Laboratory of Semiconductor Materials Science, Institute of Semiconductors, CAS, 100083 Beijing, China; 3 Physics Department, University of Konstanz, 78457 Konstanz, Germany; 4 Institute of Physics, Johannes Gutenberg-University Mainz, 55128 Mainz, Germany

## Abstract

Utilizing plasmonic nanostructures for efficient and flexible conversion of solar energy into electricity or fuel presents a new paradigm in photovoltaics and photoelectrochemistry research. In a conventional photoelectrochemical cell, consisting of a plasmonic structure in contact with a semiconductor, the type of photoelectrochemical reaction is determined by the band bending at the semiconductor/electrolyte interface. The nature of the reaction is thus hard to tune. Here instead of using a semiconductor, we employed a ferroelectric material, Pb(Zr,Ti)O_3_ (PZT). By depositing gold nanoparticle arrays and PZT films on ITO substrates, and studying the photocurrent as well as the femtosecond transient absorbance in different configurations, we demonstrate an effective charge transfer between the nanoparticle array and PZT. Most importantly, we show that the photocurrent can be tuned by nearly an order of magnitude when changing the ferroelectric polarization in PZT, demonstrating a versatile and tunable system for energy harvesting.

## Abstract (web summary)

Photoelectrochemical systems based on plasmonics require control of band bending at the interface as well as transport of hot carriers. Here, Wang *et al*. employ a ferroelectric material, Pb(Zr,Ti)O_3_, with gold on ITO to capture hot electrons from the metal and manipulate the photoexcited charges for energy conversion.

## Graphical abstract

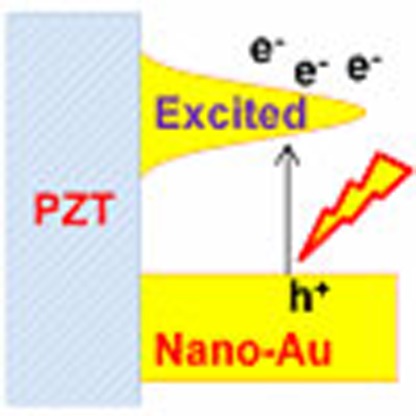

For photoelectrochemical (PEC) systems based on plasmonics, in addition to the scattering effect in metallic nanostructures^1^,^2^, three factors play decisive roles: the Schottky junction at the interface between the metallic nanoparticle and the semiconductor, enabling the capture of hot electrons generated in photon-stimulated nanometals to semicondcutors^3^,^4^,^5^; the interface between the semiconductor and electrolyte, governing the transfer of the hot carriers from the semiconductor to the electrolyte; and the transport of hot carriers between the two interfaces. Considering that the properties of the Schottky or Ohmic junction are fixed for a given combination of a metal and a semiconductor, the other two factors are crucial for adjusting the PEC performance. Particularly, the semiconductor/electrolyte interface is important, since the band bending is either upward (from semiconductor to electrolyte) for an easy hole transfer or downward to facilitate electron transfer to the electrolyte^6^,^7^. As a conventional PEC semiconductor, TiO_2_ has been widely used in water splitting for collecting hot electrons from plasmonic nanostructures^2^,^8^,^9^. However, Pt nanoparticles or other catalysts have to be adopted to adjust the upward band bending at the TiO_2_/electrolyte interface that inhibits the transfer of electrons in the conduction band of TiO_2_ to the electrolyte^10^,^11^. Though different approaches have been followed to tune the plasmonic properties of metallic nanostructures to enhance the PEC performance^12^,^13^,^14^, insightful mechanism and technique proposed for tailoring both the band bending at the semiconductor/electrolyte interface and the transport of hot carriers in the PEC film, preferably at the same time, have so far been lacking.

Here we present an approach where a conventional semiconductor has been replaced by ferroelectric Pb(Zr,Ti)O_3_ (PZT), which possesses a large, stable and manipulable remnant polarization^15^,^16^,^17^,^18^. The associated depolarization electric field (*E*_DP_), extending over the entire thin film volume, enables tuning the band bending at the ferroelectric/electrolyte interface by poling pretreatments and thus adding extra functionality for scavenging and conducting the excited charges. We report on manipulation of the charge transfer and transport in nano-Au/PZT hybrids by placing a nano-Au array in different positions within ITO/PZT and by poling the PZT films with different potentials. Among the PEC electrodes (as grown), the structure of ITO/nano-Au/PZT provides the best performance among the three structures. On the other hand, using the ITO/PZT/nano-Au/PZT electrodes, we demonstrate tuning of the short-circuit photocurrent by nearly an order of magnitude, when the pre-poling bias is switched from +10 to −10 V. The transport studies are accompanied by femtosecond transient absorbance study to track the dynamics of the hot-charge transfer from Au nanoparticles to PZT. Such simultaneous manipulation of the charge transfer and interface-related PEC phenomena within a given nano-metal/PEC film/electrolyte system presents a route to optimally and flexibly manipulate the photoexcited charges for PEC energy conversion (for example, solar water splitting).

## Results

### Characteristics of the nano-Au array

**Figure 1:**
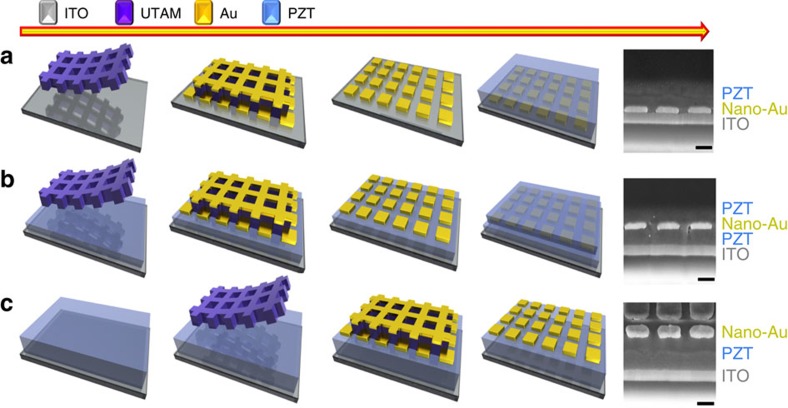
Schematic of fabrication processes for preparing the plasmonic-ferroelectric hybrids. (**a**) The procedure for fabricating the nano-Au array at the interface of ITO/PZT (structure: ITO/nano-Au/PZT). (**b**) The procedure for embedding the nano-Au array in the PZT films (structure: ITO/PZT/nano-Au/PZT), poling treatment is performed on this structure for investigating the impact of the orientation of ferroelectric polarization on the PEC performance. (**c**) The procedure for making nano-Au array on top of ITO/PZT (structure: ITO/PZT/nano-Au). Cross-sectional image of scanning electron microscope for the corresponding structure is given on the right hand. All scale bars, 200 nm.

**Figure 2:**
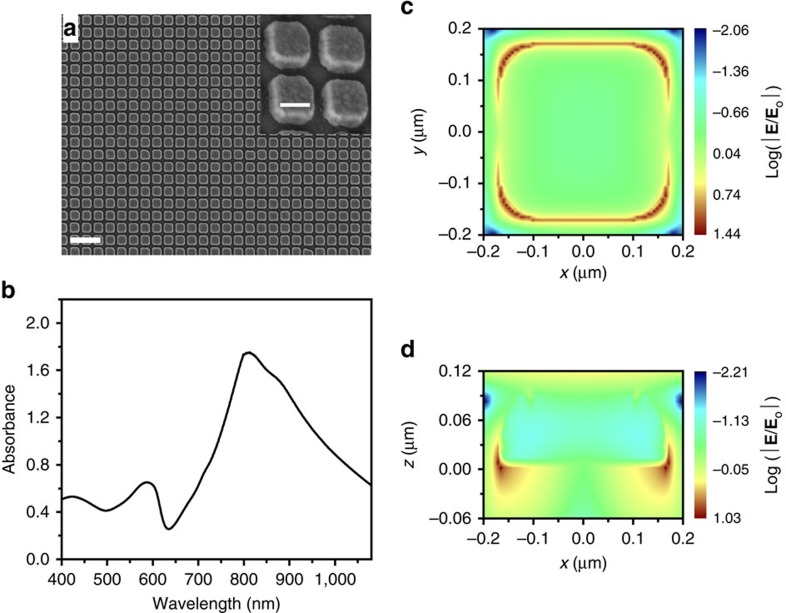
Morphology and optical absorbance of nano-Au array. (**a**) Top-view SEM images of nano-Au array fabricated on ITO glass using a UTAM template; scale bar, 1 μm. The inset zooms in one part of the SEM; scale bar, 200 nm. (**b**) Ultraviolet–visible absorbance spectrum (absorbance=−log_10_ (transmission)) of ITO/nano-Au array. (**c**,**d**) FDTD-simulated spatial distribution of the electric field intensity around the square dot ((**c**) planar FDTD; (**d**) cross-sectional FDTD) illuminated by 800 nm.

Figure 1 summarizes the fabrication processes used herein for preparing different nano-Au/PZT hybrids as PEC photoelectrodes. Well-ordered nano-Au array was prepared utilizing a well-established ultra-thin alumina mask (UTAM) technique^19^,^20^,^21^. A representative top-view scanning electron microscope (SEM) image is shown in Fig. 2a, where the spacing between the square Au dots is ∼130 nm and the dot dimension is gauged at ∼270 × 270 nm^2^ (Fig. 2a, inset). In combination with atomic force microscopic analysis illustrated in Supplementary Fig. 1, the thickness of the Au dots is determined to be ∼60 nm. These parameters govern the characteristic absorbance spectrum of the nano-Au array, as shown in Fig. 2b. The spectrum exhibits a main absorbance peak at ∼800 nm, which can be attributed to the localized surface plasmon resonance (LSPR) of the square-like Au dots with the lateral dimension of 270 nm (ref. 22). Indeed, the simulated spectra obtained by the finite difference time domain (FDTD) method, qualitatively agree well with experimental data (Supplementary Fig. 2a). Moreover, we simulated the spatial distribution of the electric field intensity around the square dot illuminated by light at 800 nm (Fig. 2c,d). The hot spots are located at the edge of the square dot and may amplify the probability of the hot-charge transfer. The advantages of such periodic nano-Au pattern are elaborated in the Supplementary Note 1 (Supplementary Fig. 3).

### Performance of plasmonic-ferroelectric hybrids

Rather than growing PZT films epitaxially in high vacuum conditions, we adopted a cost-effective spin-coating technology for obtaining high quality PZT films^18^,^23^. The X-ray diffraction patterns of the samples shown in Supplementary Fig. 4a illustrate a pure PZT phase (perovskite structure). As shown in Supplementary Fig. 4b, a typical polarization–voltage hysteresis loop yields a coercive field of ∼170 kV cm^–1^. Thus, an applied potential of 10 V is sufficient to switch the ferroelectric domains in the films of 300 nm thickness. Considering the significant role of the ITO/PZT contact on the charge transfer process, the ITO/PZT junction was elaborately investigated and the Schottky barrier was determined to be ∼1.03 eV (Supplementary Fig. 5; Supplementary Note 2).

**Figure 3:**
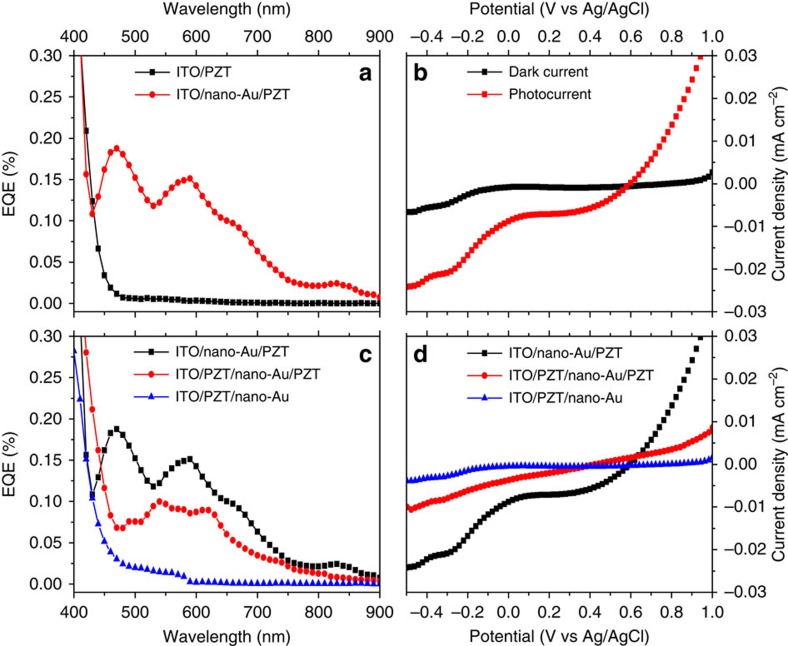
Comparison of the PEC responses from different photoelectrodes. (**a**) EQE spectra of the PEC electrodes of ITO/PZT and ITO/nano-Au/PZT, respectively. (**b**) Current density–potential curves of the ITO/nano-Au/PZT photoelectrodes under white-light excitation (filtered, *λ*>450 nm), compared with dark measurement. (**c**) EQE spectra of the electrodes of ITO/nano-Au/PZT(black), ITO/PZT/nano-Au/PZT (red) and ITO/PZT/nano-Au (blue). (**d**) Photocurrent–potential measurements of the samples in **c** under the white-light excitation (filtered, *λ*>450 nm).

Figure 3a presents the representative steady-state external quantum efficiency (EQE) spectra of PEC electrodes of ITO/PZT and ITO/nano-Au/PZT (note that the corresponding internal quantum efficiency would be at least an order of magnitude higher). Compared with the bare (intrinsic) PZT photoelectrode on ITO substrate, the nano-Au/PZT photoelectrode exhibits a distinctive EQE for photon energies below the absorption threshold of PZT (*E*_g_=3.6 eV, Supplementary Fig. 4c). The spectrum of the EQE qualitatively matches the absorbance spectrum of nano-Au/PZT (Supplementary Fig. 6a; Supplementary Note 3), demonstrating the occurrence of hot-electron injection from the excited nano-Au to PZT. Photocurrent–potential profiles were measured by soaking the photoelectrodes into 0.1 M Na_2_SO_4_ aqueous solutions. Each plot represents typical photoresponse obtained by illumination with a standard 300 W Xe lamp (Newport). To get the photocurrent signal from nano-Au array solely, a 455-nm-low pass optical filter was used to avoid the excitation of PZT. The light intensity was characterized as 100 mW cm^−2^. As illustrated in Fig. 3b, the ITO/nano-Au/PZT electrode possesses a distinct PEC performance with a short-circuit current ∼10 μA cm^−2^ and an open circuit potential close to 0.6 V versus Ag/AgCl. The photocurrent direction is cathodic, demonstrating that it is the hot electrons that have been transferred from the nano-Au to the PZT/electrolyte and hence initiate the PEC reactions. To attribute the hot-electron collection to the presence of PZT, we also made the electrode based on ITO/nano-Au and cannot observe any EQE signal at all, as shown in Supplementary Fig. 7.

Two strategies were adopted to manipulate the hot-electron injection efficiency and to optimize the PEC performance: adjusting the positions of nano-Au within the ITO/PZT and tuning the ferroelectric polarization in PZT films with external potential. First, the nano-Au array was placed in varied positions: at the interface of ITO/PZT, in the middle of PZT films and on the top of PZT films, respectively (as shown in Fig. 1). PEC results in Fig. 3c,d show that the electrodes (as grown) with nano-Au array at the ITO/PZT interface have the best performance among these three structures. The Schottky barrier of ITO/PZT is 1.03 eV (Supplementary Fig. 5). When the nano-Au array is placed in the depletion region of such Schottky contact, with continuously varying band bending, the collection and conduction of hot electrons injected into PZT should be more efficient. Importantly, the work function of Au is larger than that of ITO^24^, which supports the transfer of photogenerated holes in nano-Au into ITO. Considering the fact that the valence band position of PZT is almost 1.5 eV below the work function of Au^18^, it is on the other hand hard for the remaining holes in the Au to overcome the barrier at the Au/PZT interface and be collected by the external circuit when the nano-Au array is sandwiched within the PZT films. This can be relaxed if the ferroelectric domain structure is optimized by poling treatment^25^. As to the electrodes with the nano-Au array located on the top of the PZT, even though hot electrons can be injected into the PZT, the 1.03 eV Schottky barrier at the ITO/PZT interface prevents the electrons from being transferred to the external circuit.

**Figure 4:**
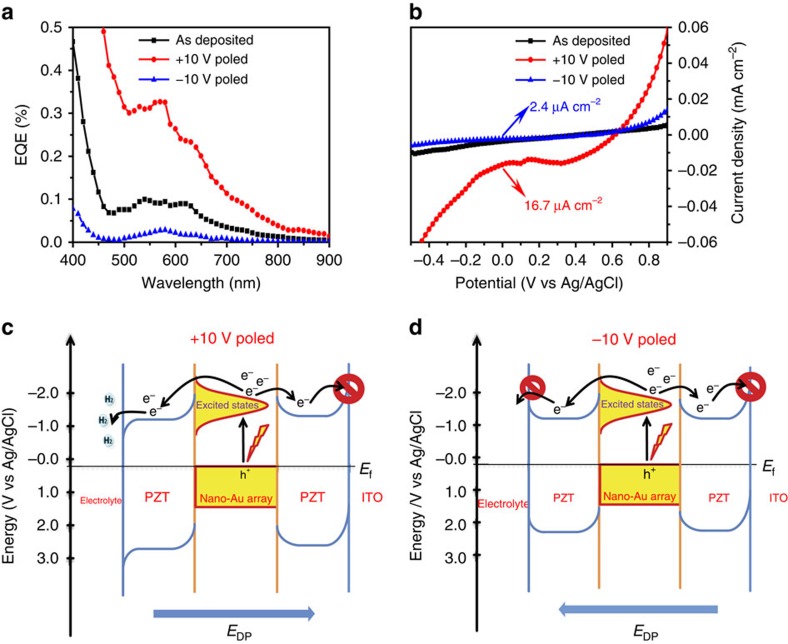
Polarization switching behavior of the ITO/PZT/nano-Au/PZT photoelectrode. (**a**,**b**) EQE spectra and photocurrent–potential measurements (under the filtered white-light excitation) of the as-grown (black), +10 V (red) and −10 V (blue) poled samples. (**c**,**d**) Schematic electronic band structure and the mechanisms for the injected hot-electron transfer from PZT films to the electrolyte for the two poling configurations.

The tunability of the *E*_DP_ in PZT films offers another opportunity to manipulate hot-electron injection and transfer. Experiments were conducted by poling the electrodes with different potentials in a propylene carbonate solution. For ITO/nano-Au/PZT, degradation of the performance was observed after poling treatments (Supplementary Fig. 8); the relevant discussion is given in Supplementary Note 4. In ITO/PZT/nano-Au/PZT electrodes, with a lower as-grown PEC performance than the ITO/nano-Au/PZT electrodes, the process was reversible and no deterioration was observed with cycling. Following poling, steady-state PEC and transient absorbance measurements were performed. Noteworthy, we demonstrate that these electrodes exhibit a high tuning capability in terms of the PEC performance. As shown in Fig. 4a, +10 V poling pretreatment results in the highest EQE compared with the same electrodes undergone –10 V poling and no poling treatments, respectively. This EQE value is even higher than that of the ITO/nano-Au/PZT electrodes, indicating that the poling condition in PZT is crucial for optimizing the PEC performance. The –10 V poling treatment strongly suppresses the EQE, while the as-grown sample shows an intermediate EQE, suggesting that the ferroelectric domains in the as-grown polycrystalline PZT films are randomly distributed. Correspondingly, the photocurrent–potential plots, displayed in Fig. 4b, demonstrate the same tendency. The short-circuit current can thus be tuned from 2.4 to 16.7 μA cm^−2^ (for white-light excitation density of 100 W cm^–2^) just by switching the poling conditions from −10 to +10 V. As shown in Supplementary Fig. 9, the poling does not affect the absorbance of this structure.

The randomly oriented ferroelectric domains in the as-grown PZT films can be poled using electric fields larger than the coercive field^26^. In this way, the direction of *E*_DP_ can be correspondingly switched^27^,^28^. The +10 V poling potential induces an *E*_DP_ with the direction pointing towards the ITO substrate and a downward band bending at the PZT/electrolyte interface (Fig. 4c). This configuration is favourable for the injected hot electrons being transferred to the interface and driving the PEC reactions. The optimized *E*_DP_ across the entire PZT films could also be helpful for transferring the excited holes to ITO electrode^25^. The –10 V poling potential, however, switches the direction of the *E*_DP_, which points towards the PZT/electrolyte interface and renders an upward band bending at the PZT/electrolyte interface (Fig. 4d). In this case, the hot electrons injected into the PZT cannot be transferred to the PZT/electrolyte interface and get trapped in the bulk of the PZT film.

We have characterized the stability of the PEC performance of our hybrid structures. As shown in Supplementary Fig. 10 and elaborated in Supplementary Note 4, the ITO/PZT/nano-Au/PZT structure shows high stability and reproducibility, indicating that the depolarization electric field in the PZT film is stable and can provide a sustainable driving force to conduct the charge carriers toward a certain direction, consistent with the previous reports^18^,^29^.

### Hot-charge transfer dynamics

To shed light on the hot-electron transfer, we performed broadband transient absorbance measurements. For photoexcitation, we used 70 fm optical pulses at 400 nm central wavelength, with the excitation density of ∼1 mJ cm^−2^. The photoexcitation photon energy (3 eV) is lower than the band gap of the PZT, yet high enough to induce the hot-electron transfer from the stimulated nano-Au to PZT. Photoinduced changes in transmission for wavelengths between 950 and 440 nm (1.3–2.8 eV) were recorded using (∼100 fs) white-light continuum pulses.

**Figure 5:**
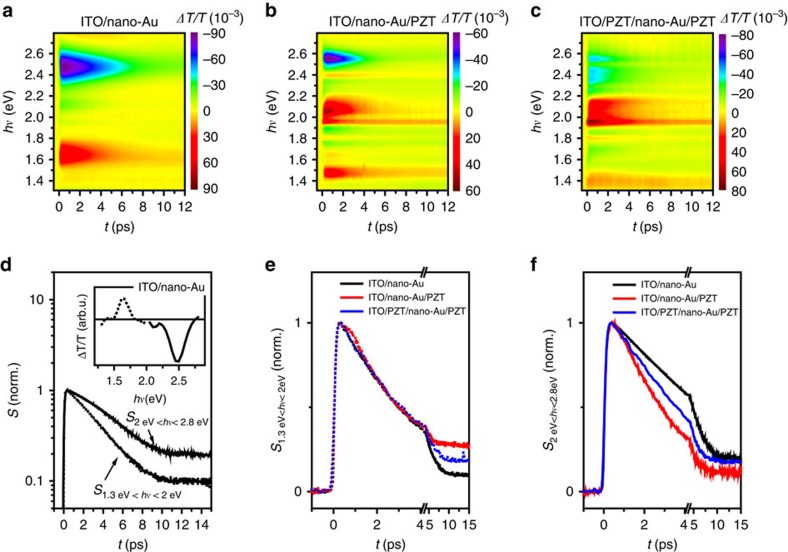
Transient absorbance spectroscopy of the plasmonic-ferroelectric hybrids. Photoinduced transmission, Δ*T/T*(*hν*,*t*) spectra recorded on (**a**) ITO/nano-Au, (**b**) ITO/nano-Au/PZT and (**c**) ITO/PZT/nano-Au/PZT, following photoexcitation with 70 fs ultraviolet pulses at 400 nm. (**d**) The time evolution Δ*T/T*(*hν*,*t*) in ITO/nano-Au that can be reproduced by Δ*T/T*(*hν*,*t*)=Δ*T/T*(1.3 eV<*hν*<2 eV) × *S*_1.3 eV<*hν*<2eV_(*t*)+Δ*T/T*(2 eV<*hν*<2.8 eV) × *S*_2 eV<*hν*<2.8 eV_(*t*). The time evolutions, *S*(*t*), are shown in the main panel, while their respective spectra are shown in inset. The dotted line presents the time evolution of the low-frequency range (1.3 eV<*hν*<2 eV), while the solid line corresponds to the high frequency (2 eV<*hν*<2.8 eV). Comparison of the low-frequency (**e**) and high-frequency (**f**) dynamics between the three nano-Au hybrids. The high-frequency response demonstrates the speeding up of relaxation in nano-Au/PZT hybrids, consistent with the existence of an additional relaxation channel (Au-PZT charge transfer).

Figure 5a presents the time evolution of the relative transmission changes (Δ*T/T*) recorded on the nano-Au array on ITO/glass. Here two distinct, spectrally well-separated components can be identified (Fig. 5d), the enhanced transmission peaked at ∼1.65 eV (∼750 nm) and the reduced transmission peaked at ∼2.5 eV. The former can be linked to the photoinduced changes in absorbance due to the photoinduced changes in the LSPR centred at ∼1.5 eV (Fig. 2b). Photoexcitation, the resulting electron–electron and electron–phonon thermalization result in broadening of the LSPR due to the enhanced scattering^30^,^31^. The strongest photoinduced changes in transmission, caused by the broadening of the LSPR, may be expected near the LSPR for photon energies where the linear transmission strongly varies with the photon energy. Apart from the broadening of the LSPR, the photoinduced shift of the central frequency may be expected due to the photoinduced expansion, particularly for longer time delays^30^.

Even more pronounced is the photoinduced decrease of transmission, peaked at about 2.5 eV. We attribute this peak to a bulk-like response of Au, governed by the photoinduced changes in the joint density of states for the optical transition between the d-band and Fermi level (*E*_f_). Indeed, the spectral shape of the induced change in transmission (Fig. 5d, inset) matches well with the results obtained on extended thin films^32^. Unlike in gold nanoparticles with lateral dimensions on the 10-nm scale, where the LSPR spectrally overlaps with the d-band to *E*_f_ transition^30^, the two spectral features are well separated in our case.

The photoinduced transmission spectra recorded on ITO/nano-Au/PZT (Fig. 5b) and ITO/PZT/nano-Au/PZT (Fig. 5c) are much more complicated. This can be linked to the complicated linear transmission spectra (Supplementary Fig. 6) caused by Fabry-Perot interference due to the additional PZT layer(s). Nevertheless, the reduced transmission in the high-frequency range and bleaching absorbance in the low-frequency part of the spectra are still recognizable. To rule out the influence of PZT on the transient dynamics in the visible range, we performed transient absorbance measurement on ITO/PZT. As shown in Supplementary Fig. 11, the contribution of PZT to the transient changes in transmission dynamics can be ignored.

Let us now address the dynamical aspect of the data. The rise-time (20−80%) is resolution limited (∼120 fs) for all samples. Plotting Δ*T/T*(*t*) in ITO/nano-Au for photon energies at the two spectral peaks we find, however, that their respective Δ*T/T* decays with considerably different time constants. To avoid artefacts that can arise due to the time-dependent spectral shifts (particularly critical for data with narrow spectral features as in Fig. 5b,c), we analysed the time evolution of the low-frequency (1.3 eV<*hν*<2 eV) and high-frequency (2 eV<*hν*<2.8 eV) responses separately. Applying singular value decomposition on both spectral ranges (Supplementary Fig. 12; Supplementary Note 5), we demonstrate that the photoinduced transient spectra in each of the spectral ranges can be well reproduced by single components (their spectral weights are ∼90% of the entire signal). In other words, the time evolution of the induced change in transmission, Δ*T/T*(*hν*,*t*), in both spectral ranges can be well reproduced by Δ*T/T*(*hν*,*t*)=Δ*T/T*(*hν*) × *S*(*t*), where Δ*T/T*(*hν*) is the spectrum and *S*(*t*) is respective temporal evolution. Figure 5d presents the decomposition of Δ*T/T*(*hν*) on ITO/nano-Au. The main panel presents the temporal evolutions for the two spectral ranges, while the inset shows the corresponding Δ*T/T*(*hν*). It should be noted that, while the two spectral ranges are Kramers–Kronig connected, the fact that the underlying excitations (LSPR and the interband transition) are well spectrally separated justifies this approach.

There are two noteworthy observations as far as the dynamics is concerned. First, the relaxation of the high-frequency part is substantially slower. Second, while the dynamics of the low-frequency part is well described by an exponential decay, the high-frequency part clearly displays a non-exponential relaxation, which can be well seen on the semi-log plot. Considering the different natures of the two processes, the observation may not be too surprising. The relaxation of the LSPR is governed by the time evolution of the (collective) plasma scattering rate, while the interband transition is governed by the photoexcited quasiparticle density and their distribution. Indeed, for the high-frequency part, the slope of *S*(*t*) changes with time, suggesting the presence of multiple decay channels^33^. Since this component corresponds to a bulk-like interband transition, the carrier diffusion (ballistic transport) into the 60-nm-thick Au nanoparticles, competing with electron–phonon thermalization, could be the origin of the observed functional form of *S*(*t*)^33^.

Further evidence for the above suggestion comes from the comparison of relaxation dynamics between different samples. While no measurable changes are observed for the low-frequency part (Fig. 5e), the high-frequency part (Fig. 5f) shows a pronounced variation of relaxation rates, recovering substantially faster in nano-Au/PZT hybrids. The relaxation rates show a clear trend: 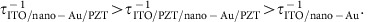 Since photoconductivity data demonstrate a substantial charge transfer from nano-Au to PZT, with the highest efficiency in ITO/nano-Au/PZT (Fig. 3c), we suggest that the nano-Au−PZT charge transfer may be responsible for speeding up the relaxation of the high-frequency spectral component in nano-Au/PZT hybrids.

We have further performed studies of photoinduced transmission on the poled ITO/PZT/nano-Au/PZT samples and some of the data (Supplementary Fig. 13) do show slight changes in the relaxation dynamics between samples poled with +10 and –10 V. While this is consist with the PEC analysis, the changes in relaxation timescales were within the scatter between timescales recorded on different (as grown) samples from the same batch.

## Discussion

Here we have studied and manipulated hot-electron transfer in nano/PZT hybrids, both from a solid-state PEC view and from the ultrafast dynamic point of view, where direct evidence of picosecond charge transfer from Au nanoparticles into PZT was obtained. To make a solid comparison of the nano-Au/PZT system with systems based on conventional semiconductors, we fabricated ITO/nano-Au/TiO_2_ photoelectrodes and tested their PEC properties. As elaborated in Supplementary Note 4, due to the n-type nature of TiO_2_ the hydrogen evolution catalyst (Pt nanoparticles) had to be deposited on TiO_2_ to adjust the band bending. The EQE of this device, which is consistent with the values summarized by Pu *et al*.^34^, is lower than in the nano-Au/PZT hybrids (Supplementary Fig. 14). After optimizing the redox couples in the electrolyte and the structural design of the plasmonic structure, the EQE of the metallic nanoparticles/semiconductor system could be enhanced to over 1% (refs 35, 36). We believe the similar could be achieved by optimizing the two factors in metallic nanoparticles/ferroelectric system.

These cumulative evidences point out that the hot-electron injection from excited nano-Au to the ferroelectric material mirrors the hot-electron transfer in nano-Au/semiconductor structures in terms of hot-electron collection. The employment of ferroelectric material in plasmonic hybrids, however, introduces another dimension to effectively manipulate the PEC properties. The tunable electric polarization offers a flexible platform to freely utilize the optical energy collected by the plasmonic nanostructure. In particular, the hot electrons could be either conducted to the ferroelectric/electrolyte interface to drive PEC reduction reactions or be transferred to the bulk of the ferroelectric material leaving the holes to initiate PEC oxidation reactions, just by switching the direction of the depolarization field in the ferroelectric films. Moreover, adding an additional finger-type electrode on top of the device would enable *in situ* control of the device performance. This concept could have a great impact on the field of solar fuel generation by water splitting or carbon dioxide reduction.

## Methods

### Fabrication of ultra-thin alumina masks and Au nanoparticle arrays

The UTAMs were prepared in a standard prepatterned anodization process. High-purity (99.99+%) aluminum foil with ∼0.22-mm thickness was used as the starting material. The aluminum foil was first degreased with acetone then annealed at 400 °C for 4 h under vacuum conditions to remove mechanical stresses. After electro-polishing in a 1:7 solution of perchloric acid and ethanol, a Ni stamp with 400 nm nanopillar array was placed on the electropolished Al foil. The pressing was carried out using an oil press system under a pressure of ∼10.0 MPa for 3 min. After that, the Ni imprinted stamp was carefully detached from the patterned Al foil and reused. The imprinting process generated an array of highly ordered concaves on the surface of the Al foil. The pre-patterned Al was anodized for 8 min under a constant voltage of 160 V in a 0.3 M phosphoric acid at 15 °C. A PMMA (poly(methylmethacrylate)) layer was subsequently deposited on the top of the alumina layer from a 6% PMMA/chlorobenzene solution to support the UTAM and baked at 100 °C for ∼25 min. The Al layer on the backside of the UTAM/PMMA was removed in a mixture solution of CuCl_2_ and HCl. The PMMA layer on the top of UTAM was removed by acetone. With the aid of a plastic strainer, the UTAM was transferred to a H_3_PO_4_ solution (5 wt %) at 30 °C for 2 h to remove the barrier layer and widen the size of the pores. Then the UTAM with uniform opened pores was transferred into deionized water from the H_3_PO_4_ solution, and the clean UTAM was carefully mounted on the substrate (ITO/glass or PZT films coated ITO/glass) in deionized water. Subsequently, the substrates with UTAMs were taken out and dried, and then Au nanoparticles were deposited into highly ordered nanopores of the UTAM by the electron beam evaporation method (Kurt J. Lesker). During the deposition process, substrates were kept in rotation at 20 rounds per minute. Finally, the UTAM was peeled off by Scotch tape, leaving a perfectly ordered nanoparticle array on the surface of the substrate.

### Preparation of polycrystalline PZT films

The PZT films with a stoichiometry of Pb(Zr_0.20_Ti_0.80_)O_3_ were deposited on ITO/glass (or ITO/glass with ordered nano-Au array) by a sol–gel method. The precursor solution for the coating was prepared by dissolving an appropriate amount of lead acetate (Pb(CH_3_COO)_2_·5H_2_O) in acetic acid at room temperature in air. A stoichiometric amount of titanium isopropoxide (Ti((CH_3_)_2_CHO)_4_) and zirconium isopropoxide (Zr((CH_3_)_2_CHO)_4_) was slowly added to the precursor solution. Subsequently, 2-methoxyethanol was added to adjust the concentration until a clear yellow sol with a molar concentration of 0.2 mol l^–1^ was obtained. A 10 mol% excess amount of lead acetate was used to compensate the Pb evaporation during annealing. The wet films were dried at 150 °C for 5 min in air and annealed at 400 °C for 10 min. Finally, the films were crystallized in air atmosphere under 550 °C for 2 h.

### Fabrication of PZT photoelectrodes

A strip of conductive copper tape was adhered on the exposed ITO part of the ITO/PZT (or ITO/nano-Au/PZT) and threaded through a glass tube, and then sealed with an insulating epoxy. Electrode areas were optically measured as 0.5 cm^2^. For measuring the hysteresis loop of the PZT films and evaluation of the Schottky barrier at the ITO/PZT, ∼40-nm-thick Pt dots with diameters of 0.28 mm were deposited onto the PZT films using physical vapour deposition.

### PEC measurements

Poling pretreatment was conducted in a quartz electrochemical cell with PZT or nano-Au/PZT photoelectrode as the working electrode and Pt plate as the counter electrode, respectively. Due to the large electrochemical windows, propylene carbonate solution containing 0.1 M LiClO_4_ was chosen as the electrolyte for poling pretreatments. The poling bias was controlled in the range between +10 and −10 V; the poling time was 10 s. Current–potential curves were measured using the digital BioLogic potentiostat (SP–200) and 0.1 M Na_2_SO_4_ aqueous solution served as the electrolyte. A Pt counter electrode and Ag/AgCl reference electrode were used during the measurements and standard 300 W Xe lamp (Newport) served as the light source; the illumination was filtered by a 455-nm filter (Newport: 20CGA–455). The intensity was 100 mW cm^−2^ determined by a Si photodiode (Newport). EQE was measured with an Oriel 150 W Xe arc lamp (Newport) and a quarter-turn single-grating monochromator (Newport). Measurements were recorded with chopped illumination (20 Hz) and no external bias was applied during the measurements to get a pure photocurrent signal. The output current signal was connected to a Merlin digital lock-in radiometry system and the output signal from the lock-in amplifier was fed into a computer controlled by TRACQ BASIC software.

### Characterizations

X-ray diffraction measurement was recorded on Bruker D8 Advance equipped with graphite monochromatized high-intensity Cu Kα radiation (*λ*=1.54178 Å). The SEM images were obtained by Auriga Zeiss focused ion beam SEM. Transient absorbance spectroscopy was carried out by 70 fs optical pulses at 400 nm central wavelength, with the excitation density of 1 mJ cm^−2^. The wavelength of the pump pulse was selected as 400 nm to avoid exciting PZT. Room-temperature ultraviolet–visible absorbance spectroscopy was carried out on Varian Cary 5,000 UV–vis-NIR spectrophotometer. Polarization–voltage hysteresis loops were examined using a precision ferroelectric analyzer from Radiant Technology. The dark leakage current–voltage (*J*–*V*) curves of ITO/PZT/Pt were recorded by Keithley 4,200. FDTD simulations were performed using FDTD Solutions by Lumerical Computational solutions, Inc. Atomic force microscopic measurements were carried out using NTEGRA Probe NanoLaboratory (NT-MDT).

## Additional information

**How to cite this article:** Wang, Z. *et al*. Manipulation of charge transfer and transport in plasmonic-ferroelectric hybrids for photoelectrochemical applications. *Nat. Commun.* 7:10348 doi: 10.1038/ncomms10348 (2016).

## Supplementary Material

Supplementary Information Supplementary Figures 1 - 14 and Supplementary Notes 1 - 5.
