# Supplementary material for: Manipulation of charge transfer and transport in plasmonic-ferroelectric hybrids for photoelectrochemical applications
Source: Nat Commun. 2016 Jan 12;7:10348. doi: 10.1038/ncomms10348 (PMC4729958; doi:10.1038/ncomms10348)
Supplement: Supplementary Information — Supplementary Figures 1 - 14 and Supplementary Notes 1 - 5. [file ncomms10348-s1.pdf]

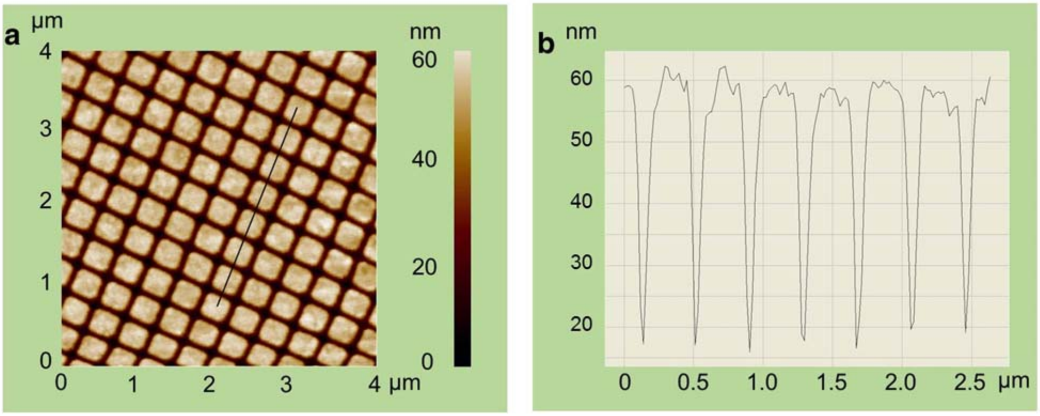

Supplementary Figure 1. AFM analysis of nano-Au array.

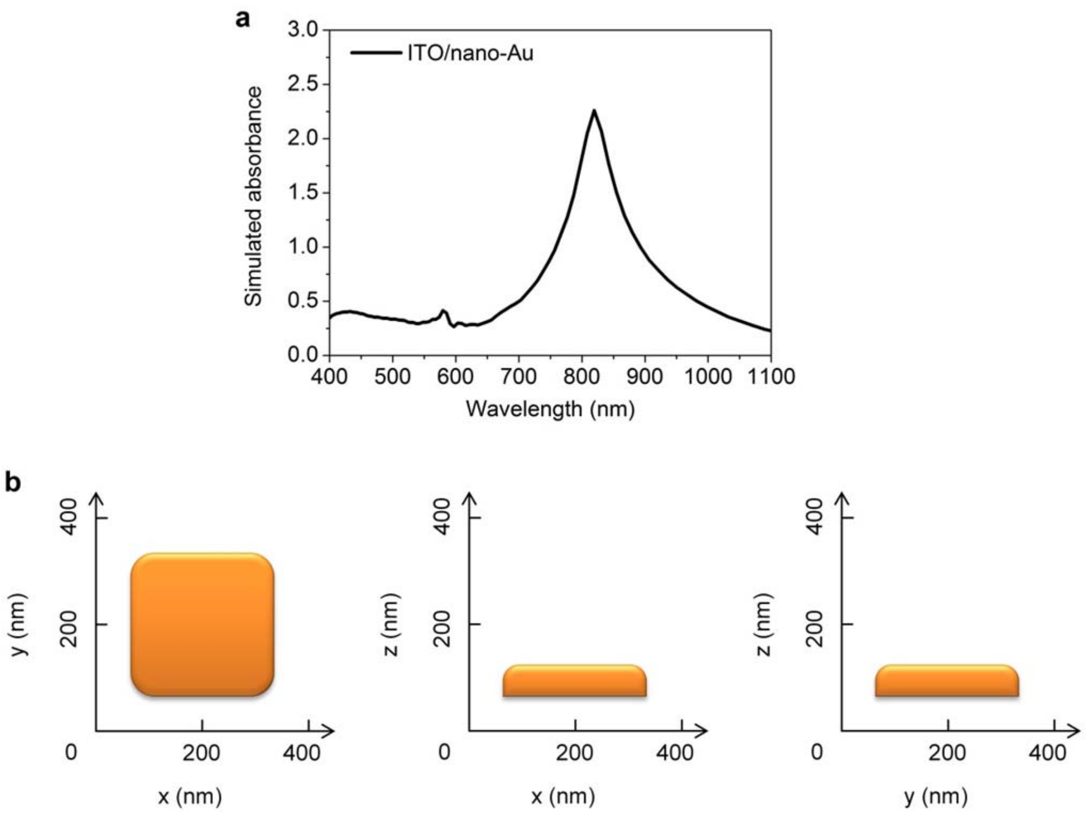

Supplementary Figure 2. Absorbance simulation. (**a**) FDTD simulated absorbance spectrum of the ITO/nano-Au. (**b**) Sketches of the nano-Au morphology used in simulation.

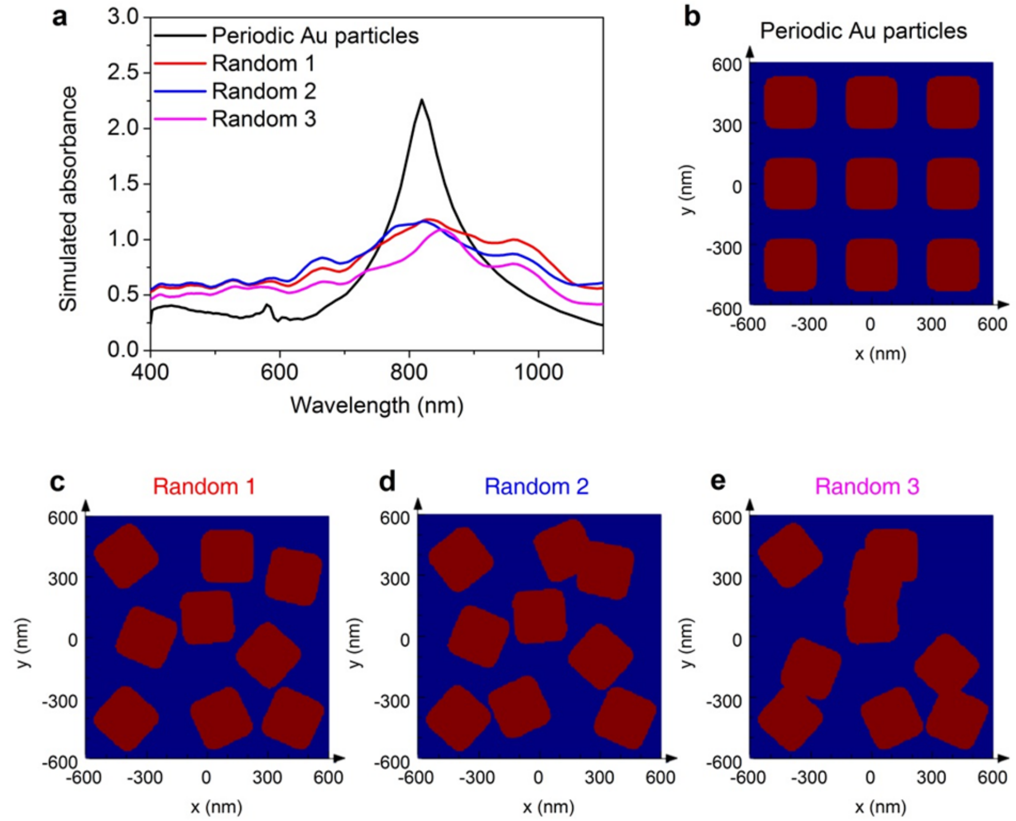

Supplementary Figure 3. Simulation of absorbance for different morphologies. (**a**) Simulated absorbance spectra of the periodic Au particle array (black) compared to the absorbance of randomly distributed Au particle films (three different structures were simulated: red, blue and magenta). The schematics of the structures are shown in (**b**,**c**,**d**,**e**).

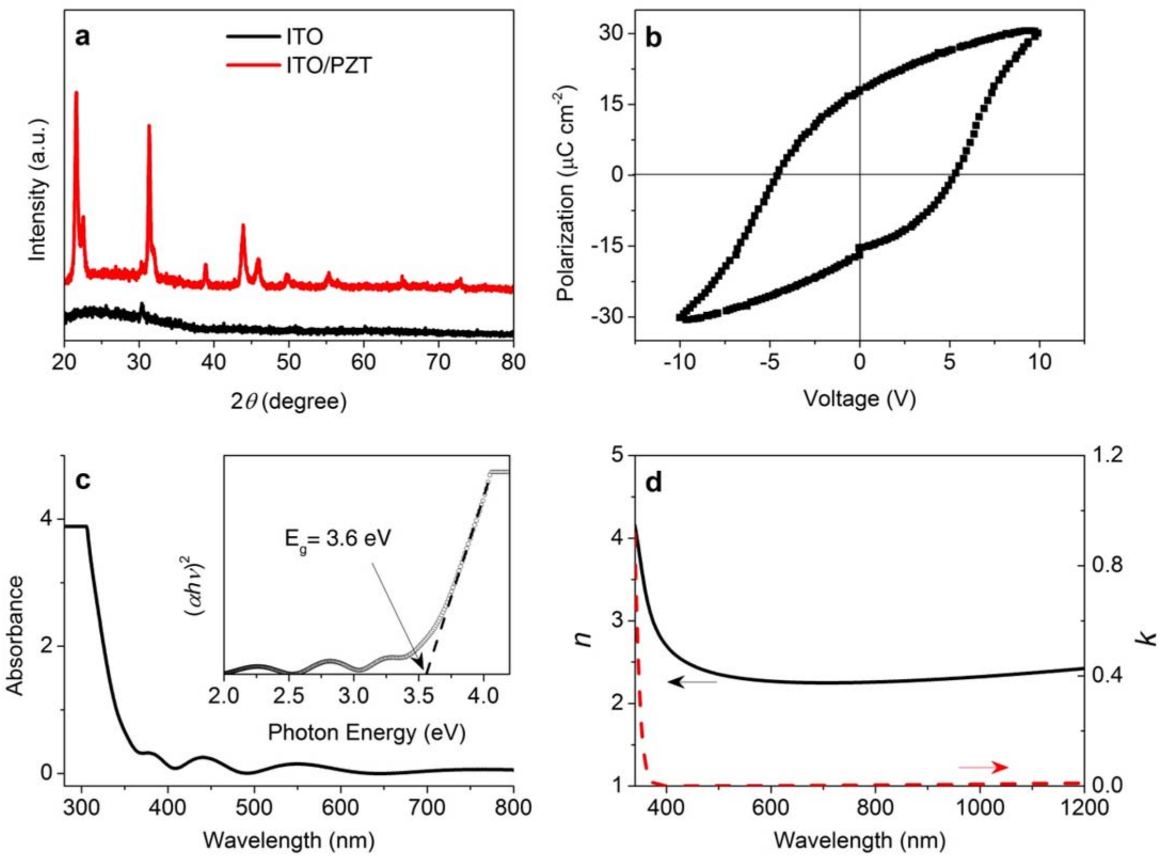

Supplementary Figure 4. Characterizations of PZT films. (**a**) XRD patterns of the prepared PZT films on ITO/glass. (**b**) Polarization–voltage hysteresis loop measurements of the PZT films. (**c**) Absorbance analysis of the PZT films on ITO/glass. (**d**) Wavelength-dependence of the measured refractive index and extinction coefficient of polycrystalline PZT films on ITO glass.

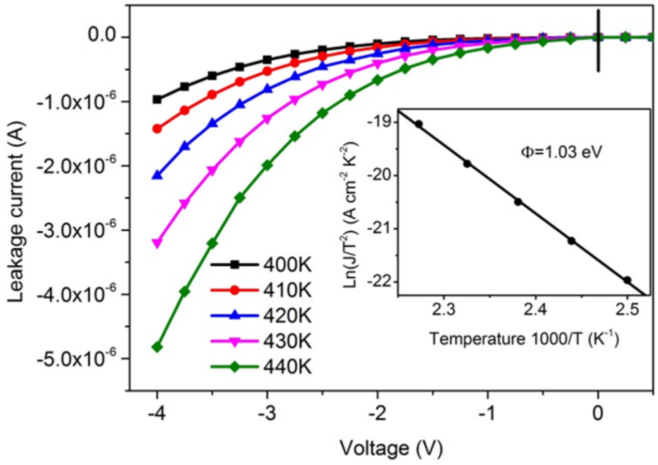

Supplementary Figure 5. Dark leakage current–voltage plots for the structure of ITO/PZT/Pt. Inset: the fitting of Schottky–Simmons equation.

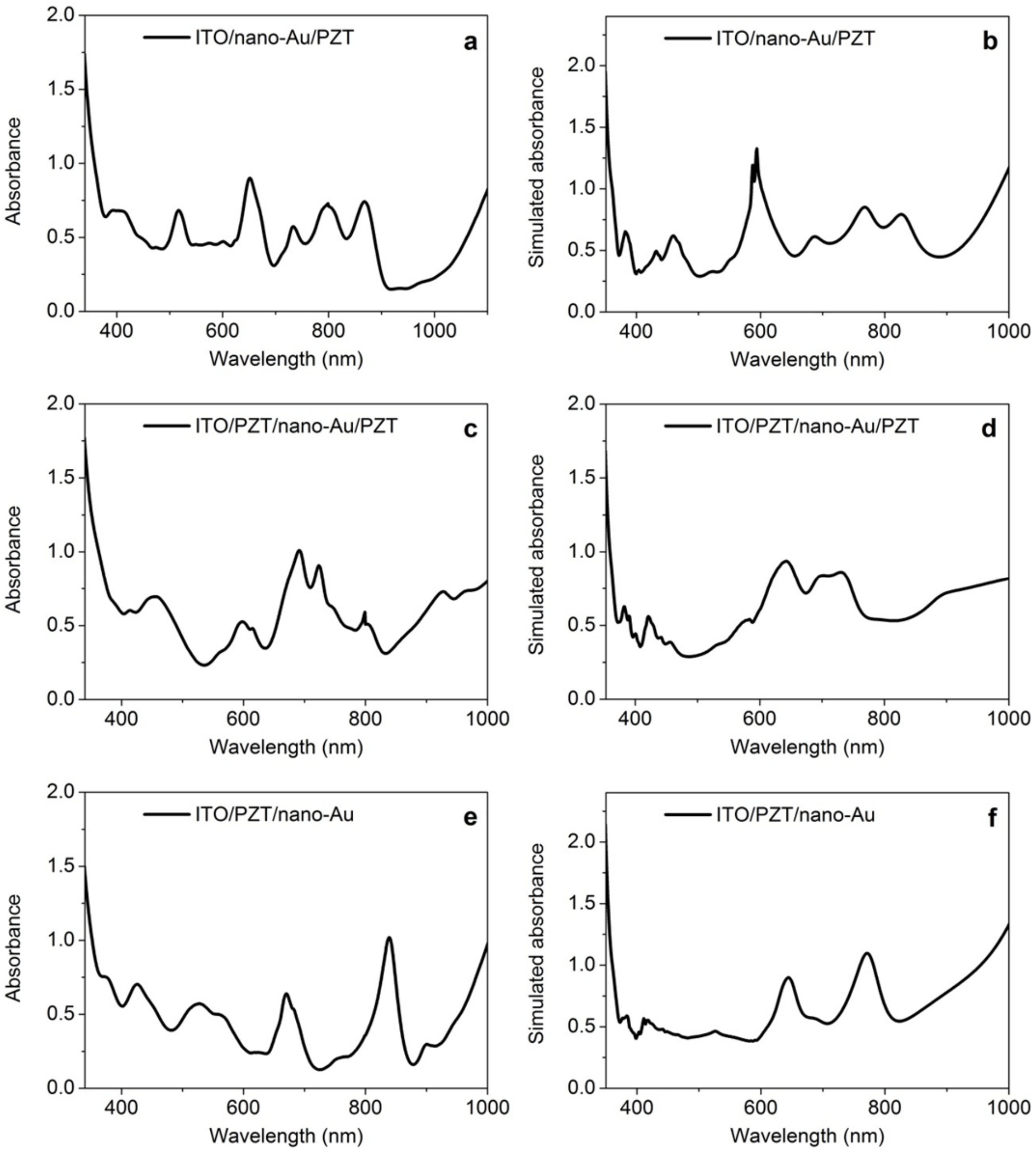

Supplementary Figure 6. Comparison of the absorbance spectra of different photoelectrodes. Measured absorbance spectra (a, c, d) compared to the FDTD simulated spectra (b, d, f) of the ITO/nano-Au/PZT, ITO/PZT/nano-Au/PZT and ITO/PZT/nano-Au, respectively.

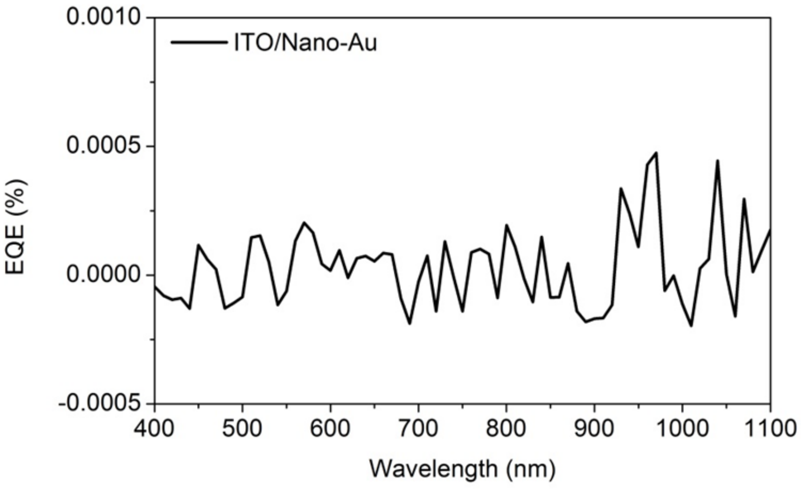

Supplementary Figure 7. EQE spectra of the ITO/nano-Au electrode.

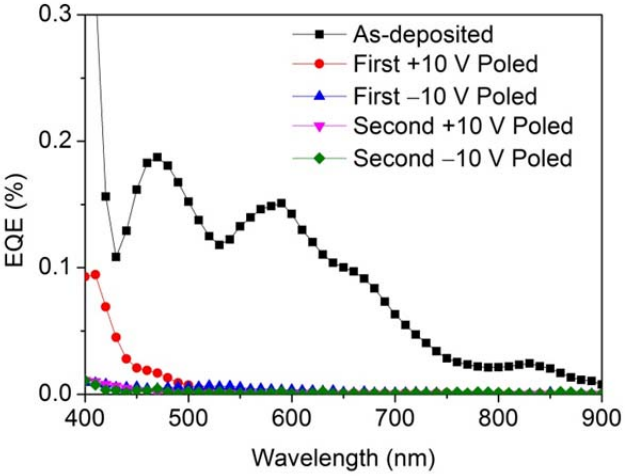

Supplementary Figure 8. EQE spectra of the ITO/nano-Au/PZT electrode under different conditions.

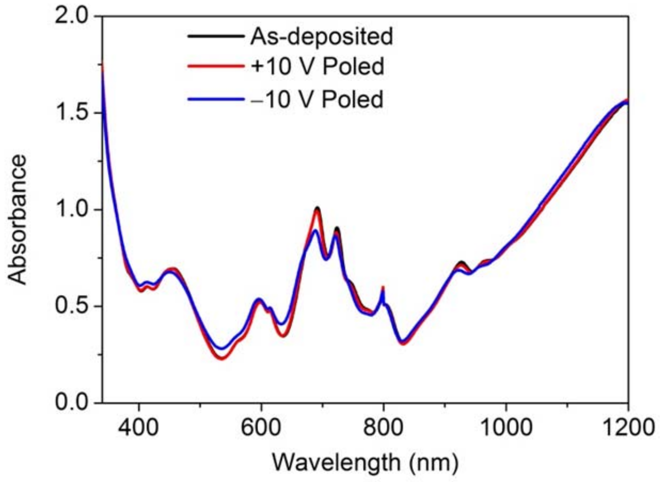

Supplementary Figure 9. Absorbance spectra of ITO/PZT/nano-Au/PZT under different poling conditions: as-grown (black), +10 V (red) and –10 V (blue).

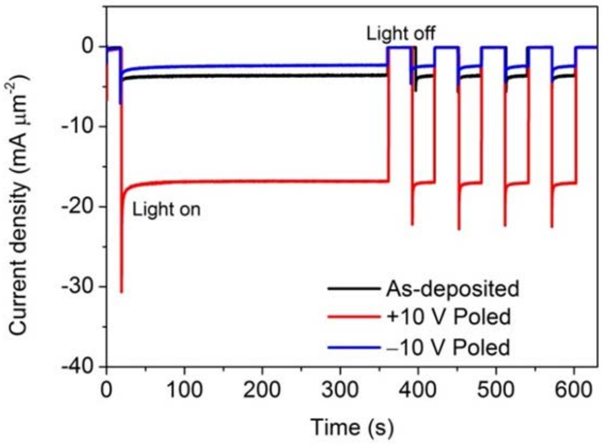

Supplementary Figure 10. Stability of ITO/PZT/nano-Au/PZT under different poling conditions. Time-dependent short circuit photocurrent (for white light excitation density of 100 W cm^{-2}) for sample ITO/PZT/nano-Au/PZT in three states: as-deposited and after +10 V and –10 V poling. The PZT film was poled in the propylene carbonate solution containing 0.1 M $LiClO_4$ by applying a voltage of +10 V or –10 V between the photoelectrode and the Pt counter electrode.

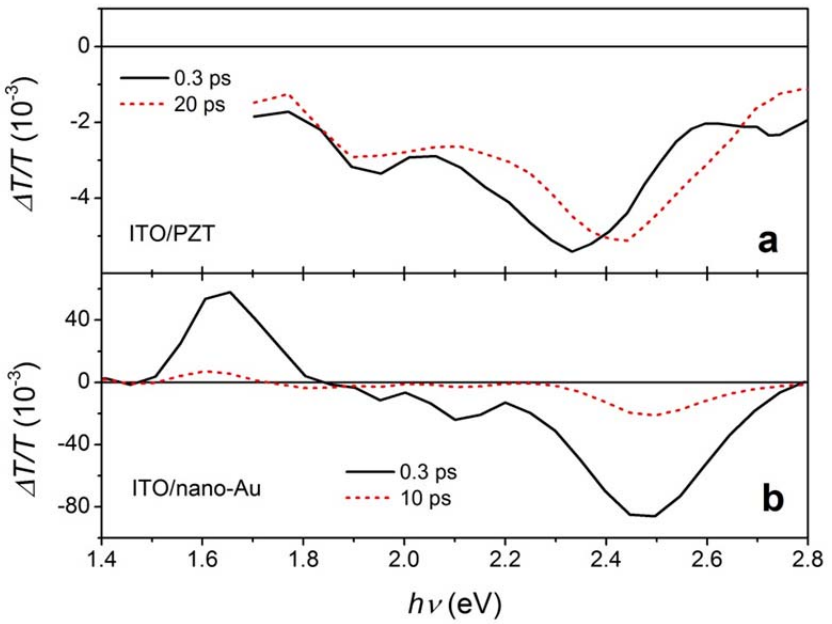

Supplementary Figure 11. Transient absorbance in bare PZT films. Spectra of the photoinduced change in transmission at different time-delays (see labels) recorded on (a) the bare PZT film on ITO-glass substrate and (b) on ITO/nano-Au. The data were recorded at comparable excitation densities. Note that the induced changes in transmission in ITO/PZT are about 20-times lower, displaying only weak changes with increasing the time-delay.

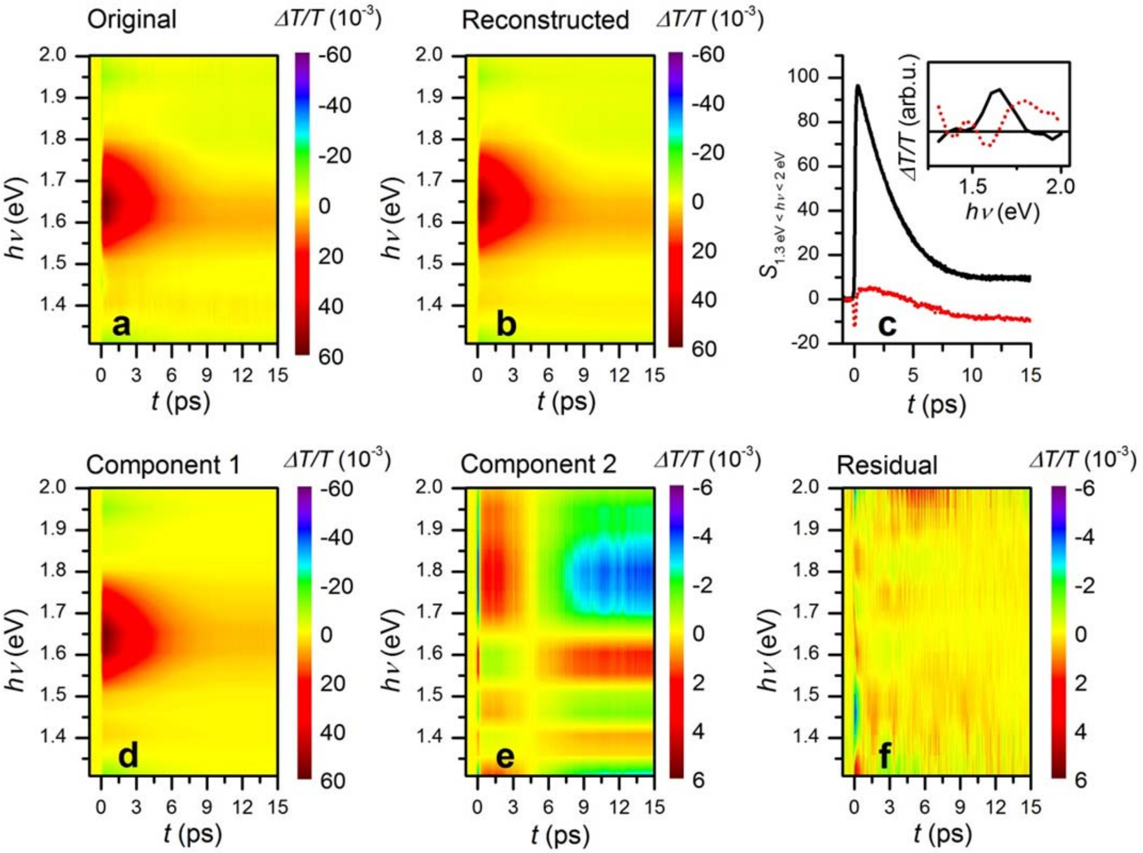

Supplementary Figure 12. Singular value decomposition analysis of ∆*T/T*(1.3 − 2 eV) traces, recorded on ITO/nano-Au.

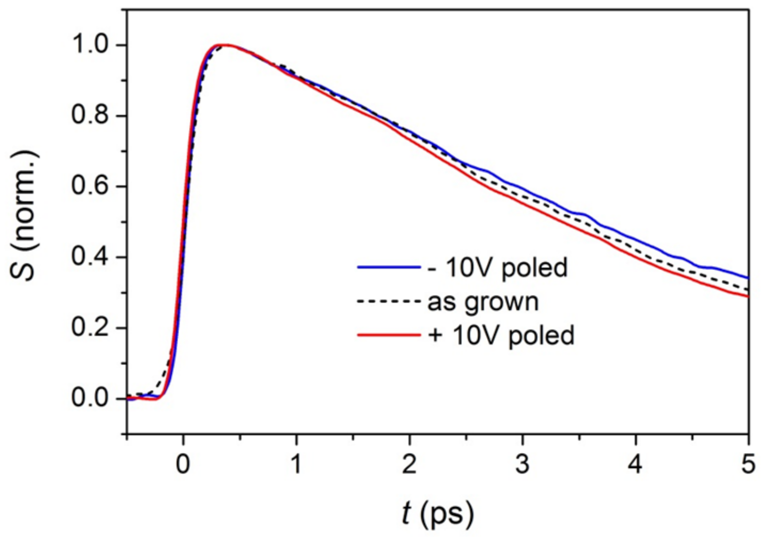

Supplementary Figure 13. Normalized transient absorbance traces (integrated high frequency response) of the sample ITO/PZT/nano-Au/PZT before poling (dashed black curve) and after +10 V (red) and –10 V (blue) poling treatments.

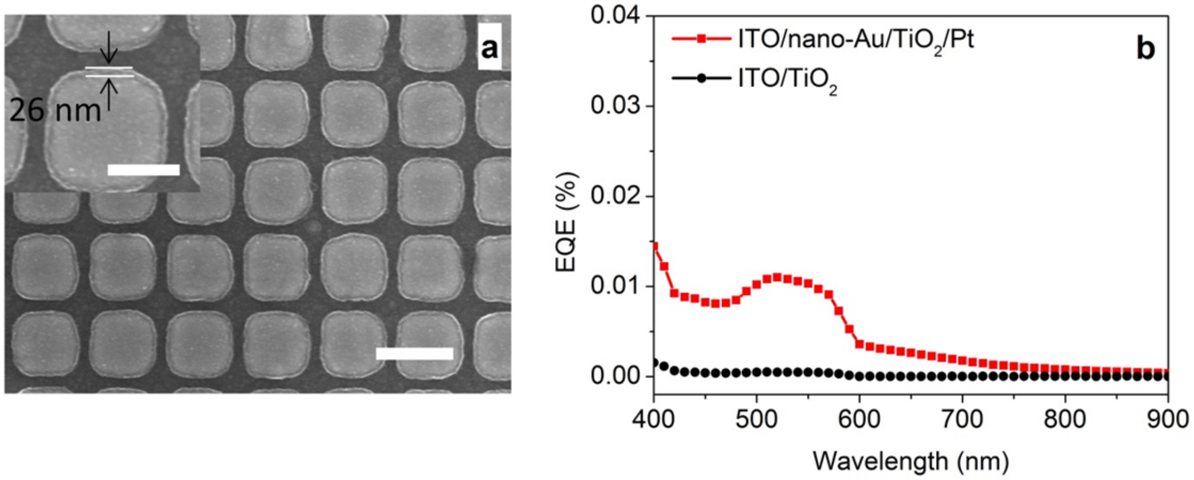

Supplementary Figure 14. Performance of ITO/nano-Au/TiO_2. (**a**) Top-view SEM images of nano-Au nanoparticle arrays after coating with a thin shell of TiO_2 with a thickness of ~26 nm by ALD, scale bar, 400 nm (inset scale bar: 200 nm). (**b**) EQE spectra of the PEC electrodes of ITO/TiO_2 and ITO/nano-Au/TiO_2/Pt, respectively.

Supplementary Notes

Supplementary Note 1. The characterizations of nano-Au array and the reasons for using periodic plasmonic nanostructures.

To supplement the morphology characterization of the prepared nano-Au array, atomic force microscopic (AFM) was utilized. The relevant results are presented in Supplementary Fig. 1. Identical parameters to the ones obtained by the SEM measurements were retrieved. Importantly, the thickness was gauged as 60 nm, providing a set of accurate parameters for the FDTD simulation. Supplementary Fig. 2a presents the absorbance spectrum of the ITO/nano-Au structure obtained by the FDTD method. The absorbance is in a good agreement with the experimental data (see Fig. 2b in the main paper), showing a well-defined LSPR near 800 nm. To simulate the absorbance of the PEC electrodes, we used the geometrical parameters of the nano-Au arrays that closely match the ones of the real samples. The parameters for individual nanoparticles are shown in Supplementary Fig. 2b.

The reasons for introducing periodic plasmonic nanostructures. (1) Periodic nano-Au array with well controlled geometrical parameters provides a good platform to investigate the absorbance properties and charge transfers of the system both experimentally and theoretically. (2) The geometrical parameters are well defined. The control over the shape, size, distance between nanoparticles and thickness of the ferroelectric layer enables control over the absorption and thus provides further optimization tools. Therefore periodic patterned structures are necessary for further optimization. (3) To directly demonstrate the advantages of the periodic patterns, we have performed a simulation to show how linear absorbance is affected by randomness. As shown in Supplementary Fig. 3, the film with periodic nano-Au array has a much higher LSPR absorbance as in the case nanoparticles being randomly oriented. Moreover, the resonance is much sharper. (4) In conventional films containing Au nano-particles, which are usually prepared by spin-coating or spay-coating the solution with Au particles, the distribution of the plasmonic particles is almost impossible to control. It is unavoidable to have some aggregations of particles. Such aggregations affect the absorbance, and could give rise to hot-spots, and making the whole structure also mechanically fragile.

Supplementary Note 2. Characterizations of the prepared PZT polycrystalline films

Supplementary Fig. 4a presents the XRD pattern of the PZT film on ITO coated glass substrate. The three main diffraction peaks could be ascribed to the reflection of (001), (101) and (002) planes of the PZT (JCPDS card No.70–4260), respectively, indicating the pure perovskite structure. Polarization–voltage (P–V) hysteresis loop, shown in Supplementary Fig. 4b, demonstrates the ferroelectric properties of PZT. The coercive field of about 170 kV cm^{-1} can be directly estimated from the hysteresis loop of the 300 nm thick film. Thus a 10 V poling voltage is sufficient for turning the ferroelectric domains. Supplementary Fig. 4c presents the absorbance spectra of the prepared PZT films. A band gap value of 3.6 eV can be extracted by plotting $(\alpha h\nu)^2$ vs $h\nu$ (inset) and determining the intercept of the linear slope with the x-axis. The extracted optical band gap of 3.6 eV matches the values reported elsewhere[1,2]. Below the absorbance threshold the interference fringes can be observed in PZT films.

Determination of the Schottky barrier height at ITO/PZT. The dark current–voltage (J–V) characteristic of ferroelectric films is related to the Schottky barrier height. The Schottky emission can be expressed by[1,3,4]*:*

$$J = A^*T^2\exp\left(\frac{-q(\Phi-\sqrt{qE_m/4\pi\varepsilon_r\varepsilon_0})}{kT}\right) \quad , \tag{1}$$

where A^*, Φ, ε_0, and ε_r are the effective Richardson constant, potential barrier height, permittivity of free space, and dynamic dielectric constant, respectively. E_m is the maximum electric field at the Schottky interface. Equation (1) can be converted in the following way to simplify further analysis:

$$\ln\left(\frac{J}{T^2}\right)=\ln(A^*) - \frac{q}{kT}\,\Phi + \frac{q}{kT}\sqrt{qE_m/4\pi\varepsilon_r\varepsilon_0} \tag{2}$$

Given that E_m is proportional to the applied voltage V, $\ln\left(\frac{J}{T^2}\right)$ vs $V^{1/2}$ plots should be linear for a constant T. The intercept at V=0 yields the value:

$$\Psi = \ln\left(\frac{J}{T^2}\right)_{V\to 0}=\ln(A^*) - \frac{q}{kT}\,\Phi \tag{3}$$

From equation (3), Φ could be easily extracted, since Ψ depends linearly on 1/T. Supplementary Fig. 5 shows the J–V characteristics of ITO/PZT/Pt in the temperature range between 400 and

440 K. This temperature range above 400 K was used, for the trapped carriers to be sufficiently activated to enable the current to be stable during the measurement. Since the Pt/PZT contact analysis is not relevant for the discussion, we just present the part of the J–V curves from ITO/PZT contact. Plotting Ψ vs 1/T, the Schottky barrier height of the ITO/PZT interface can be extracted (inset to Supplementary Fig. 5). The extracted value of 1.03 eV is in good agreement with the reported values[1,5].

Supplementary Note 3. Optical properties of plasmonic-ferroelectric hybrids in equilibrium

To simulate the ITO/nano-Au/PZT structures, we first measured the refractive index and extinction coefficient (*n* & *k*) values of our PZT films. The data are presented in Supplementary Fig. 4d. These values match qualitatively the reported ones[6], the slight difference can be ascribed to the interface reactions of ITO/PZT during the annealing process. Supplementary Fig. 6 presents the absorbance spectra of our hybrid structures with nano-Au placed at different positions within the ITO/PZT multilayer. The spectra differ considerably from the spectra of the pure ITO/nano-Au structure. This is due to the change of the dielectric surroundings of nano-Au and the Fabry-Perot interferences. The simulated spectra of relevant structures are presented in the right panels, demonstrating a qualitative agreement with the measured ones.

The absorbance spectra of ITO/PZT/nano-Au/PZT structure under different poling conditions. To make a convincing conclusion that the poling influences only the charge transfer between Au and PZT, we measured the absorbance spectra of ITO/PZT/nano-Au/PZT structure under various poling conditions. As shown in Supplementary Fig. 9, the changes in the equilibrium absorbance are marginal, comparable to the variation of absorbance between different samples within the same batch. These results indicate that poling does not impact the band structure of PZT or LSPR properties of the nano-Au.

Supplementary Note 4. Photoelectrochemical properties of PEC electrodes

PEC performance for the ITO/nano-Au. Supplementary Fig. 7 presents the measurement of the EQE performance of the ITO/nano-Au. Within the error bars the EQE is zero in this energy range. ITO is a degenerate semiconductor and behaves as a conductor to collect the photo-generated charge carriers. Once ITO is contacted to the nano-Au, there is no band bending at the interface

to collect the hot electrons generated in plasmonic nanoparticles. Thus there is no measurable PEC response.

Poling effect and the performance of the ITO/nano-Au/PZT photoelectrode. Supplementary Fig. 8 shows the EQE spectra of the ITO/nano-Au/PZT electrode. Without the poling treatment, the electrode shows an EQE of ~0.2%, higher than that of the ITO/PZT/nano-Au/PZT electrode. However, after poling treatment, the nano-Au/PZT film was found to deteriorate, demonstrated by the data shown in Supplementary Fig. 8. In fact, after poling treatment small pieces of film even peeled off. Therefore, a reliable comparison of the device performance for different poling conditions could not be performed. For the ITO/PZT/nano-Au electrode, even though hot electrons can be injected from excited nano-Au to the PZT, the Schottky barrier at the ITO/PZT hinders the electrons from being collected by the ITO electrode. Correspondingly, no EQE signal from the hot electron injection was observed. Since the Schottky barrier cannot be changed reversely by the poling treatment, it is meaningless to study the poling effect on the performance of this electrode. Therefore, we only investigate the influence of poling on the PEC performance of ITO/PZT/nano-Au/PZT electrodes.

The stability of the PEC performance of the photoelectrode ITO/PZT/nano-Au/PZT under different poling conditions. Supplementary Fig. 10 presents the photocurrent vs time for the ITO/PZT/nano-Au/PZT electrode under different poling conditions. The photocurrent was collected at the bias of 0 V vs Ag/AgCl. After turning on the photoexcitation, the current rapidly increases, followed by a rapid decay within 10 s. After 15 s, the photocurrent becomes stable and its value can be maintained for a long time. The behavior is similar to the case of PEC studies in conventional semiconductors[7]. Noteworthy, the photocurrent under +10 V poling is always higher than that from the same electrode without the poling treatment or under −10 V poling. The difference between the three photocurrents do not change with time, indicating the stable domain structure of the PZT film. As such it can provide a sustainable driving force to transfer the photo-generated charge carriers toward a certain direction, consistent with previous reports[1,8]. Moreover, considering the stable depolarization field, the screening charges at the surface of the ferroelectric material can extend over considerable distances from the surface. This is further beneficial for conducting the photo-generated charges to the surface and to drive the PEC reactions. Similarly, the screening charges could also increase the photocurrent of the electrode/ferroelectric/electrode structure, as reported by Qin et al. [9].

The PEC performance of the ITO/nano-Au/TiO_2 electrode. The ITO/nano-Au/TiO_2 structure was fabricated using a similar method as the one used to prepare the ITO/nano-Au/PZT, except that

the TiO_2 was synthesized by a standard atomic layer deposition procedure (ALD)[10]. The corresponding SEM image is shown in Supplementary Fig. 14a. Here the nano-Au array is the same to that in the ITO/nano-Au/PZT hybrids, while the thickness of the TiO_2 was 26 nm. The thickness of the TiO_2 was chosen to be considerably lower as in the case of PZT. A thicker TiO_2 layer would prevent the transfer of electrons to the electrolyte, since, due to the n-type nature of TiO_2, the band bending at the TiO_2/electrolyte promotes the conduction of holes to the electrolyte. Only after the hydrogen catalysts (Pt nanoparticles) are deposited on TiO_2, which modify the band bending, could the photo-generated electrons be extracted to the electrolyte. As shown in Supplementary Fig. 14b, the EQE is lower than that of ITO/nano-Au/PZT structure, not to mention the lack of charge transfer tuning capability of the PZT films. These results further evidence the advantages of using ferroelectric materials for energy harvesting in plasmonic hybrid nanostructures.

Supplementary Note 5. Analysis of the transient absorbance data

To demonstrate that the transient absorbance data describe the carrier dynamics and transport from nano-Au, we performed control transient absorbance studies on bare PZT films deposited on ITO-glass. Supplementary Fig. 11 presents the comparison of the data recorded on ITO/PZT (panel a) with the data on ITO/nano-Au (panel b). The induced changes in bare PZT are about 20-times smaller than in ITO/nano-Au on ITO/nano-Au/PZT hybrids (note that both, signal magnitudes and time-scales are comparable in samples containing nano-Au). Moreover, the dynamics in ITO/PZT is step-like, with slow relaxation, probably governed by self-trapping on defects.

Spectral decomposition by means of singular value decomposition. To determine the time evolution of excitations, that give rise to the recorded differential transmission transients, we performed singular value decomposition (SVD) analysis on the data. Since the data reveal different timescales for the low and high frequency ranges, the analysis was performed separately on the two spectral ranges. We should note that the analysis is not affected substantially when shifting the border between the high and low frequency ranges by 0.1 eV. Supplementary Fig. 12 presents the analysis of the differential transmission transients, $\Delta T/T(h\nu,t)$, in the range between 1.3 and 2 eV (low frequency range).

SVD is a mathematical method which in our case decomposes the data into several spectral components, with each of them described by an individual time trace. In the Supplementary Fig.

12 panel a) presents the original data, while panel b) presents the reconstructed part, where two main components, whose time traces and their corresponding spectra are shown in panel c) and its inset, respectively. The two spectral components are separately shown in panels d) and e) (note the scale change in panel e), while the residual (the difference between the original data) and the reconstructed data are plotted in panel f).

It follows that $\Delta T/T(h\nu,t)$ can be well approximated by a single component (Component 1, discussed in the main text). The spectral shape of Component 2 looks like the spectral derivative of Component 1. Its time evolution (displaying a buildup on the same timescale as component 1 is decaying), and the fact that its spectral weight is only about 10% of the entire spectral weight, suggest that it corresponds to the lattice heating effect. Similar results were obtained also for the high frequency range, as well as for ITO/nano-Au/PZT and ITO/PZT/nano-Au/PZT samples.

Poling effect on the transient absorbance recovery time for the structure of ITO/PZT/nano-Au/PZT. To study the effect of poling, we performed photoinduced transmission measurements on the sample ITO/PZT/nano-Au/PZT using different poling conditions. Supplementary Fig. 13 presents the time evolution of the integrated response on as grown, as well as +10 V and –10 V poled samples. One can recognize the trend suggesting that recovery proceeds slightly faster in the sample poled with +10 V. On the other hand, the difference in timescales is rather weak, comparable to the variation between timescales obtained on different samples of the same batch. As demonstrated in the main text, the +10 V poling potential induces a depolarization electric field ($\mathbf{E_{DP}}$) with the direction pointing towards the ITO substrate and a downward band bending at electrolyte/PZT interface, which is favorable for the injected hot electrons being transferred to the interface and driving PEC reactions. The –10 V poling potential, however, switches the direction of the $\mathbf{E_{DP}}$. In this case, the injected hot electrons in PZT cannot be transferred to the PZT/electrolyte interface and are trapped in the bulk of the PZT film. As the hot electrons aggregate in the PZT films, the hot electrons injection rate could be slowed down, giving rise to an increase in the recovery time as opposed to the +10 V pre-poled samples.

Supplementary References

1. Cao, D. *et al.* High-efficiency ferroelectric-film solar cells with an n-type Cu_2O cathode buffer layer. *Nano Lett.* **12,** 2803–2809 (2012).

2. Majumder, S. B., Mohapatra, Y. N., Agrawal, D. C. Optical and microstructural characterization of sol – gel derived cerium-doped PZT thin films. *J. Mater. Sci.* **32,** 2141–2150 (1997).

3. Qin, M., Yao, K., Liang, Y. C. & Gan, B. K. Stability of photovoltage and trap of light-induced charges in ferroelectric WO_3-doped $(Pb_{0.97}La_{0.03})(Zr_{0.52}Ti_{0.48})O_3$ thin films. *Appl. Phys. Lett.* **91,** 092904 (2007).

4. Pintilie, L., Vrejoiu, I., Hesse, D., LeRhun, G. & Alexe, M. Ferroelectric polarization-leakage current relation in high quality epitaxial $Pb(Zr,Ti)O_3$ films. *Phys. Rev. B* **75,** 104103 (2007).

5. Zhang, P. *et al.* Enhanced photocurrent in $Pb(Zr_{0.2}Ti_{0.8})O_3$ ferroelectric film by artificially introducing asymmetrical interface Schottky barriers. *Mater. Chem. Phys.* **135,** 304–308 (2012).

6. Zheng, F. *et al.* Photovoltaic enhancement due to surface-plasmon assisted visible-light absorption at the inartificial surface of lead zirconate-titanate film. *Nanoscale* **6,** 2915–21 (2014).

7. Chen, Y.-S. & Kamat, P. V. Glutathione-capped gold nanoclusters as photosensitizers. Visible light-induced hydrogen generation in neutral water. *J. Am. Chem. Soc.* **136,** 6075–82 (2014).

8. Wang, C. *et al.* Photocathodic behavior of ferroelectric $Pb(Zr,Ti)O_3$ films decorated with silver nanoparticles. *Chem. Commun.* **49,** 3769–3771 (2013).

9. Qin, M., Yao, K. & Liang, Y. C. Photovoltaic mechanisms in ferroelectric thin films with the effects of the electrodes and interfaces. *Appl. Phys. Lett.* **95,** 022912 (2009).

10. Marichy, C., Bechelany, M. & Pinna, N. Atomic layer deposition of nanostructured materials for energy and environmental applications. *Adv. Mater.* **24,** 1017–1032 (2012).
